# Supplementary material for: Unraveling the role of urea hydrolysis in salt stress response during seed germination and seedling growth in Arabidopsis thaliana
Source: eLife. 2024 Jul 22;13:e96797. doi: 10.7554/eLife.96797 (PMC11364434; doi:10.7554/eLife.96797)
Supplement: Figure 2—figure supplement 1—source data 1. — Genomic DNA was extracted from young leaves of WT and transformed plants, and amplified by PCR using primers flanking the target sites to confirm the introduction of mutations. The PCR products were sequenced to identify double mutants, atargah1/atargah2. The primer sequences were using primers AtArgAH1-FW/RV and AtArgAH2-FW/RV (Supplementary file 1). Inset bases in the atargah1/atargah2 mutant sequence are highlighted in yellow. [file elife-96797-fig2-figsupp1-data1.docx]

WT>AT4G08900 ArgAH1

AATGGGTTTGAAGCACGTCTTGATTCTGAATTATGGTTTCTGTTGTTATTGGATCACCTTGCTTGTGCTGTTCTATGGTTTATACAGTCATTGATGATTGGTTTTGACCTTTGTGTTTGGTAGACTTGTTTGTCTTTTGATATTGATCTGTTGAATGAATTGCAGGGAAGGAGTTGAAGGATCCACGGGTTCTAACTGATGTTGGGGATGTTCCGGTACAAGAGATTAGAGATTGTGGGGTTGATGATGATAGACTGATGAATGTCATAAGTGAATCTGTGAAGTTGGTGATGGAAGAGGTAAAGCTATTTGACAGCTCTAGTGTTACGCCACTTCATGTATTTAGTCTTTTGTGAATCTTCC

*argah1/argah2*>AT4G08900 ArgAH1

CCCCTCGCTTGAAGCACAGTCTTGATTCTGAATTATGGTTTCTGTTGTTATTGGATCACCTTGCTTGTGCTGTTCTATGGTTTATACAGTCATTGATGATTGGTTTTGACCTTTGTGTTTGGTAGACTTGTTTGTCTTTTGATATTGATCTGTTGAATGAATTGCAGGGAAGGAGTTGAAGGATCC**C**ACGGGTTCTAACTGATGTTGGGGATGTTCCGGTACAAGAGATTAGAGATTGTGGGGTTGATGATGATAGACTGATGAATGTCATAAGTGAATCTGTGAAGTTGGTGATGGAAGAGGTAAAGCTATTTGACAGCTCTAGTGTTACGCCACTTCATGTATTTAGTTCTTTTGTGAATTCCCCG

WT>AT4G08870 ArgAH2

GTCACGTAGAGCGTGTCTTGATGCTTCGTTAACTCTCATCCGTGAAAGGGCAAAACTCAAAGTATATACATTGTTTTATTAAGGCTTATCATCTAATTAAAGTTTTCTTTTCTTTCTGTAATATGAAATAAATAGATTATTGTATTTGTATGTATTGTTTTAGGGAGAGTTAGTGCGACTCATAGGAGGAGCAAAAGCTACAACAGCTCTTCTTGGAGTACCACTTGGTCACAACTCTTCTTTTCTTGAAGGCCCAGCCTTGGCTCCTCCTCATGTAAGGGAAGCTATTTGGTGTGGTAGTACAAACTCCACCACTGAAGAAGGTATAACAAAACTTGTATTTAGAATGTTGGACAGTTTCATGTTTTGTATAGCAACAATCTAGCTGATAATATTTGTTTTTTTACTTATTATTACTCAATAAGT

*argah1/argah2*>AT4G08870 ArgAH2

AATTCACGGCGACGTGTCTTGATGCTTCGTTAACTCTCATCCGTGAAAGGGCAAAACTCAAAGTATATACATTGTTTTATTAAGGCTTATCATCTAATTAAAGTTTTCTTTTCTTTCTGTAATATGAAATAAATAGATTATTGTATTTGTATGTATTGTTTTAGGGAGAGTTAGTGCGACTCATAGGAGGAGCAAAAGCTACAACAGCTCTTCTTGGAGTA**C**CCACTTGGTCACAACTCTTCTTTTCTTGAAGGCCCAGCCTTGGCTCCTCCTCATGTAAGGGAAGCTATTTGGTGTGGTAGTACAAACTCCACCACTGAAGAAGGTATAACAAAACTTGTATTTAGAATGTTGGACAGTTTCATGTTTTGTATAGCAACAATCTAGCTGATAATATTTGTTTTTTTACTTATTATTACTCAATAAGATCCCGGTTTATAAA
