# Supplementary figures and images for: Unraveling the role of urea hydrolysis in salt stress response during seed germination and seedling growth in Arabidopsis thaliana
Source: eLife. 2024 Jul 22;13:e96797. doi: 10.7554/eLife.96797 (PMC11364434; doi:10.7554/eLife.96797)

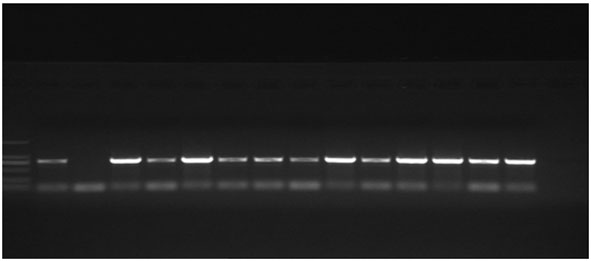

Supplement: Figure 7—figure supplement 1—source data 1. — The AtDur3 gene was amplified using the specific primers AtDur3-FW and AtDur3-RV. [file elife-96797-fig7-figsupp1-data1.zip › Figure 7 - supplement 1 - source data 1.jpg]

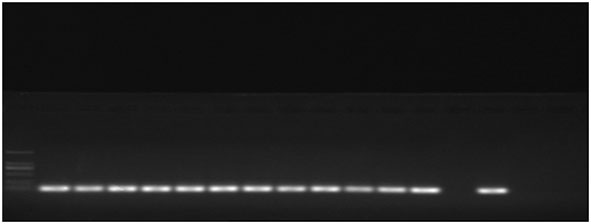

Supplement: Figure 7—figure supplement 1—source data 2. — The Actin gene was amplified using the primers AtActin-FW and AtActin-RV. [file elife-96797-fig7-figsupp1-data2.zip › Figure 7 - supplement 1 - source data 2.jpg]

*AtDur3*


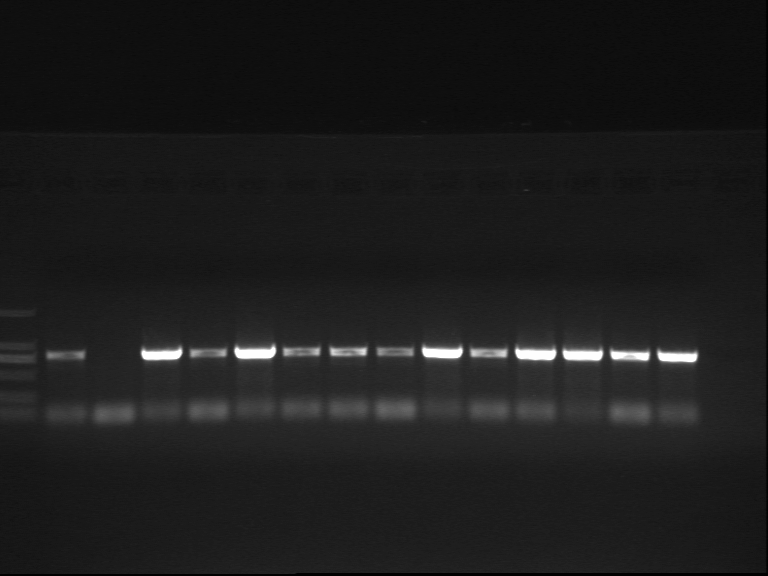


2000bp

1000bp

750bp

500bp

250bp

100bp

WT

*atdur3*

*Actin*


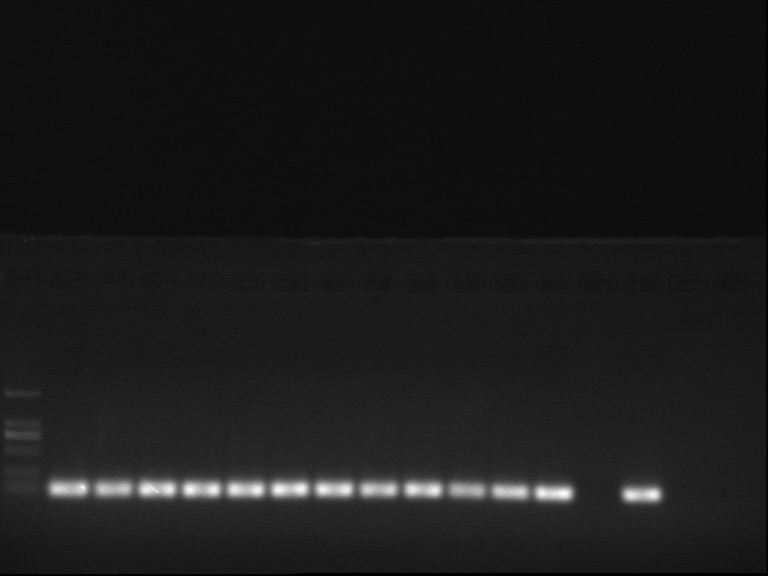


1000bp

2000bp

750bp

500bp

100bp

250bp

WT

*atdur3*

Supplement: Figure 7—figure supplement 1—source data 3. — Total RNA was extracted from young leaves of WT and atdur3 plants. Gene-specific primers pairs AtDur3-FW and AtDur3-RV were used for AtDur3, while AtActin-FW and AtActin-RV were used for Actin (Supplementary file 1). Actin expression was used as an internal control for normalization of the RT-PCR expression data. [file elife-96797-fig7-figsupp1-data3.docx]
