# Supplementary material for: Unraveling the role of urea hydrolysis in salt stress response during seed germination and seedling growth in Arabidopsis thaliana
Source: eLife. 2024 Jul 22;13:e96797. doi: 10.7554/eLife.96797 (PMC11364434; doi:10.7554/eLife.96797)
Supplement: Supplementary file 1. — The T-DNA insertion homozygous lines SALK_057987 (atargah1) were amplified using primers 057987-LP or 057987-RP and a T-DNA primer LBP. The AtDur3 gene was amplified using the specific primers AtDur3-FW and AtDur3-RV. The Actin gene was amplified using the primers AtActin-FW and AtActin-RV. The PCR products of double mutants atargah1/atargah2 were sequenced using primers AtArgAH1-FW/RV and AtArgAH2-FW/RV. The PRpHluorin gene was amplified using the specific primers PRpHluorin-F and PRpHluorin-R. [file elife-96797-supp1.docx]

**Supplementary File 1**

**Supplementary Table 1. PCR primer sequences used in the current study.**

| **Primer** | **Sequence (5’- 3’)** |
| --- | --- |
| LBP  057987-LP  057987-RP  AtArgAH1-FW  AtArgAH1-RV  AtArgAH2-FW  AtArgAH2-RV | ATTTTGCCGATTTCGGAAC  AACTATTTGCGTATTTGATCCG  AGCTTCTCGAATACGAGGAGG  ACATGGGTTTCATTATGAAC  CACAAAAGACTAAATACATG  CCTTGCGGTCCTTGCCAAC  ATAAACAGAATCTTATTGAG |
| AtDur3-FW  AtDur3-RV  AtActin-FW  AtActin-RV  PRpHluorin-F  PRpHluorin-R | CTCTTTACATATGACATCTACCGAAC  TATCTTCTCAGCTTCTGGCACAG  GGTAACATTGTGCTCAGTGGTGG  AACGACCTTAATCTTCATGCTGC  GGGGGATCCATGAGTAAAGGAGAAGAACTTTTCACT CGCCTCGAG TTATTTGTATAGTTCATCCATGC |
